# Newborn Boys and Girls Differ in the Lipid Composition of Vernix Caseosa

**DOI:** 10.1371/journal.pone.0099173

**Published:** 2014-06-09

**Authors:** Radka Míková, Vladimír Vrkoslav, Robert Hanus, Eva Háková, Zuzana Hábová, Antonín Doležal, Richard Plavka, Pavel Coufal, Josef Cvačka

**Affiliations:** 1 Institute of Organic Chemistry and Biochemistry, Academy of Sciences of the Czech Republic, Prague, Czech Republic; 2 Department of Analytical Chemistry, Faculty of Science, Charles University in Prague, Prague, Czech Republic; 3 3rd Internal Department, General Faculty Hospital and 1st Faculty of Medicine, Charles University in Prague, Prague, Czech Republic; 4 Department of Obstetrics and Gynaecology, General Faculty Hospital and 1st Faculty of Medicine, Charles University in Prague, Prague, Czech Republic; Cnrs France

## Abstract

Vernix caseosa protects the skin of a human fetus during the last trimester of pregnancy and of a newborn after the delivery. Besides its cellular and proteinaceous components, an important constituent and functional agent is a complex lipid fraction, implicated in a multitude of salubrious effects of vernix caseosa. Little is known about how the chemical composition of vernix caseosa lipids is affected by various biological characteristics of the baby, such as the gestational age, birth weight, and, last but not least, the gender of the newborn. This study reports on the chemical variability of lipids contained in the vernix caseosa of twenty newborn girls and boys and shows that the quantitative patterns of the lipids are sex-specific. The specificity of lipids was investigated at the level of fatty acids in the total lipid extracts and intact lipids of several neutral lipid classes. Hydrocarbons, wax esters, cholesteryl esters, diol diesters and triacylglycerols were isolated using optimized semipreparative thin-layer chromatography, and the molecular species within each class were characterized using matrix-assisted laser desorption/ionization mass spectrometry. Statistical evaluation revealed significant quantitative sex-related differences in the lipid composition of vernix caseosa among the newborns, pronounced in the two lipid classes associated with the activity of sebaceous glands. Higher proportions of wax esters and triacylglycerols with longer hydrocarbon chains were observed in newborn girls.

## Introduction

Vernix caseosa (VC) is a white creamy substance which coats the skin of a human fetus and of a newborn [1] and which is produced during the third trimester of gestation [2]. In utero, it serves as a waterproofing film and modulator of transepidermal water flux [3], facilitates the final stages of the skin and gastrointestinal system development and protects the skin from some of the agents present in amniotic fluid [4]. After the birth, it acts as an antibacterial shield [5], [6] and helps the neonate to adapt to the dry environment [7]. Very low birth-weight preterm infants lack VC and are susceptible to invasive infections because of insufficient formation of the stratum corneum [8], [9]. The skin of prematurely born babies suffers from excessive water loss, resulting in dangerous dehydration and heat loss [10], [11]. VC also shows a remarkable ability to enhance wound healing, which promises new therapies for patients with altered skin integrity after burn injuries or skin diseases. Because a therapeutic use of native VC from mature newborns is impossible, clinically relevant artificial substitutes of VC are to be developed [12], [13].

VC is a complex biofilm composed of water in hydrated corneocytes (80%), surrounded by a matrix of lipids (10%) and proteins (10%) [1], [2]. The lipid fraction is extremely rich and not yet fully characterized despite the efforts of numerous researchers [14]–[19]. The most abundant lipid classes (wax esters – WE, cholesteryl esters – CE, diol diesters – DD and triacylglycerols – TG) are known, but they are characterized typically only with respect to their fatty acids (FA) composition, investigated from hydrolyzed lipid fractions.

Virtually nothing is known about the chemical variability of VC lipids depending on the gestational age and health conditions, the changes in the chemical composition during fetal development or the possible diagnostic value of VC components. All this information is of importance for current neonatology and medicine in general. Sex-related aspects of the early skin development are not well understood either. Certain differences between VC lipids of newborn boys and girls were reported in early eighties [15], [20] using analytical methodology available at that time and a limited number of samples. The data relied either on semi-quantitation of lipid classes separated by thin layer chromatography (TLC) or lipid hydrolysis followed by analysis of fatty acid methyl esters (FAME). The structures of intact lipids involved in sex-related differences have not been disclosed. Recent advances in analytical instrumentation, namely in mass spectrometry, allow us to have a closer look at the chemistry of vernix caseosa and the human skin ontogeny from a different perspective.

Matrix-assisted laser desorption/ionization mass spectrometry (MALDI MS) is a powerful tool in protein and peptide analytics, increasingly utilized also in lipidomics [21]–[24]. The method allows intact lipids to be detected without previous modification and may yield quantitative results [25]. Modern MALDI MS setups also make it possible to fragment selected peaks, e.g., by tandem time-of-flight (TOF/TOF) instrumentation and thus to obtain more detailed structural information [22]–[26].

In this paper, we investigate sex-related differences in the lipid composition of VC in twenty newborn boys and girls at the level of FAME and intact, non-hydrolyzed lipids using MALDI MS. Since the cutaneous barrier formation and sebaceous gland activity are controlled by sex hormones [27]–[29], we test a hypothesis that the composition of VC lipids is gender-related. For this purpose, we have developed a method for a detailed characterization of intact lipids in VC. The lipids were isolated, separated into neutral lipid classes and the molecular species within the lipid classes were analyzed using MALDI-TOF MS and MALDI-TOF/TOF MS. The resulting data were statistically evaluated with respect to the sex specificity.

## Materials and Methods

### Chemicals

Analytical-grade hexane, chloroform, diethyl ether, acetone and ethanol were purchased from Merck (Darmstadt, Germany) or Penta (Chrudim, Czech Republic) and distilled in glass before use. Chloroform was stabilized with 1% of ethanol. Gradient-grade methanol was bought from LachNer (Neratovice, Czech Republic). 2,6-Di-terc-butyl-4-methylphenol (BHT), Florisil^®^ for TLC and acetyl chloride were obtained from Fluka (Buchs, Switzerland). Magnesium sulfate (p.a.), polyethylene glycols (PEG, reagent-grade), primuline and rhodamine 6G were purchased from Sigma-Aldrich (St. Louis, MO, USA). Silica gel 60 G with gypsum (12%) was obtained from Merck and silver carbonate was from Lachema (Brno, Czech Republic). Deionized water was manufactured by the Milli Q system (Millipore, Milford, MA, USA). Lipid standards (99% purity) were bought from Sigma-Aldrich (squalene - SQ, stearyl behenate), Larodan (Malmö, Sweden; cholesterol – Chol, tristearin, distearin and palmitolein), Nu-Chek Prep (Elysian, MN, USA; stearic acid) and Matreya LLC (Pleasant Gap, PA, USA; phosphatidylcholine). MALDI-TOF MS matrices were supplied by Fluka (2,5-dihydroxybenzoic acid – DHB; 2-mercaptobenzothiazole – MBT; 7,7,8,8-tetracyanoquinodimethane – TCNQ; 4-nitroaniline – 4NA; picolinic acid – PA) and Sigma-Aldrich (2,4,6-trihydroxyacetophenone – THAP). The sodium salt of 2,5-dihydroxybenzoic acid (NaDHB) and the lithium salt of 2,5-dihydroxybenzoic acid (LiDHB) were synthesized and prepared as described previously [26].

### Sample collecting

Healthy male (10) and female (10) subjects (Table S1) delivered at full term were included in this study. VC samples (1–2 g) were collected immediately after the delivery into glass vials and stored at −25°C. The exact location of sampling (back, buttocks, groins, legs, arms) varied depending on the VC layer thickness. Blood-contaminated samples were discarded. The samples were collected with written informed parental consent and the work was approved by the Ethics Committee of the General University Hospital, Prague (910/09 S-IV); the study was performed according to the Declaration of Helsinki.

### Isolation of lipids and their TLC separation

The VC samples were suspended in 50 ml of chloroform:methanol 2∶1 (V/V) with 0.05% BHT. The suspension was cleared of epithelial cells by filtration through a column containing purified cotton-wool and silica gel (60–120 µm, ca 0.2 g). Anhydrous MgSO_4_ (ca 5 g) was added to absorb water, and the suspension was filtered again. The solvents were removed by a rotary evaporator (35°C, 170 mbar) and a stream of argon. The isolated lipids were stored in glass vials at −25°C.

The lipids (ca 20 mg) were separated on 9×12 cm glass TLC plates coated with silica gel using hexane:diethyl ether (93∶7, V/V) as a mobile phase. Each plate was developed twice to focus the zones (in the first step to 3/4 of the plate height and then, after air-drying, to the top). The zones were visualized under UV light after being sprayed with rhodamine 6G (0.05% in ethanol); an example of the thin layer chromatogram is shown in Figure S1. The zones corresponding to particular lipid fractions (classes) were identified using standards and published data [19] as follows: SQ (R_f_ 0.89–0.94), WE + CE in one zone (R_f_ 0.66–0.74), DD (R_f_ 0.46–0.52), TG (R_f_ 0.19–0.27), free fatty acids - FA (R_f_ 0.10–0.13), Chol (R_f_ 0.06–0.08) and highly polar lipids (R_f_ 0.00–0.01). Only neutral lipids (SQ, WE, CE, DD and TG) were further isolated and analyzed in this study. Each zone was scratched off into a column with purified cotton-wool and silica gel; neutral lipids were eluted using diethyl ether. The solvent was evaporated under a stream of argon; the separated lipids were dissolved in chloroform:methanol 2∶1 (V/V, 1 mg/ml) and stored at −25°C.

Due to their similar polarities, WE did not separate from CE on silica gel sorbents; their separation required magnesium-based materials to be used [30], [31]. Therefore, we separated WE (R_f_ 0.54–0.68) from CE (R_f_ 0.32–0.48) using 20×10 cm glass TLC plates coated with Florisil (activated magnesium silicate) with a hexane:diethyl ether (90∶10, V/V) mobile phase [32]. The plates were activated at 120°C for 1 h before the separation. The zones were visualized using primuline in methanol:water 1∶1 (V/V) under UV radiation (366 nm). WE and CE were extracted from the plates as described above.

### Transesterification and GC/MS of FAME

Total lipid extracts of VC were transesterified using a method described by Stránský and Jursík [33]. Briefly, lipids were dissolved in chloroform:methanol (2∶3, v/v) in a small glass ampoule. After adding acetyl chloride, the ampoule was sealed and placed in a water bath at 70°C. After 60 min the ampoule was opened, the reaction mixture was neutralized with silver carbonate and injected onto GC column. FAME were analyzed using a 7890N gas chromatograph (Agilent, Santa Clara, CA, USA) coupled to a 5975C quadrupole mass spectrometer and equipped with a fused silica capillary column DB-wax (30 m×0.25 mm, 0.25 µm, J&W 122-7032). The carrier gas was helium at 1.5 mL/min. The injector was held at 250°C and operated with a split ratio of 1∶20; 2 µL of sample solution (chloroform:methanol (2∶3, v/v)) was injected. The temperature program: 140°C (0 min), then 5°C/min to 250°C (50 min); total run time was 72 min. 70 eV EI mass spectra were recorded in the mass range of 25–600 u; 3 min solvent delay was used. Temperatures of the transfer line, ion source and quadrupole were 250°C, 230°C and 150°C, respectively. The chromatographic peaks representing FAME were identified based on the presence of *m/z* 74 and *m/z* 87 in their mass spectra. FAME were relatively quantified from their peak areas integrated in the total ion current chromatograms.

### MALDI MS

MALDI-TOF MS measurements were performed on a Reflex IV (Bruker Daltonik GmbH, Bremen, Germany) operated in the reflectron mode with an acceleration voltage of 20 kV and an extraction pulse of 200 ns. A nitrogen UV laser (337.1 nm, a 4 ns pulse of 300 µJ, a maximum frequency of 20 Hz) was utilized for desorption and ionization. Matrix ions were suppressed below *m/z* 200. The mass spectra were externally calibrated using PEG oligomers. The MS spectra were averaged from 1,000 laser shots collected at various places across the spot. Fragmentation was performed using ultrafleXtreme equipped with smartbeam laser (Bruker Daltonik GmbH, Bremen, Germany). A MS/MS LIFT method for small molecules mode with an ion source and LIFT acceleration voltage set to 7.5 kV and 19 kV, respectively was utilized for the fragmentation. Precursor ions were selected by ion selector mass window ±1 Da. The spectra were averaged from at least 20,000 shots. The data were collected and processed using FlexAnalysis 3.0 or 3.3 (Bruker Daltonik GmbH).

The choice of the matrix is crucial for successful MALDI MS. Therefore, a study was undertaken to select suitable matrices for lipid classes studied in this work. Because of the neutral character of the analytes lacking easily ionizable groups, matrices permitting ionization via metal-ion attachment were needed. The matrices were selected based on 1/their ability to ionize the analytes at low laser fluencies, 2/the absence of analyte-fragment ions in the spectra, 3/the simplicity of the isotope clusters, and 4/the low interference of the matrix background ions with analyte signals. The investigated matrices were prepared as saturated solutions in the solvents specified in Table S2 and co-deposited with the samples on the MALDI plate (MTP 387-position ground steel target; Bruker Daltonik GmbH) by mixing the sample with the matrix before application (CE, DD, TG) or by covering the matrix with the sample (WE).

In agreement with previous findings [26], LiDHB, providing [M+Li]^+^ adducts, proved to be the most suitable matrix for SQ and WE. The same matrix appeared to work well also for CE and DD; LiDHB was found to be more suitable for CE than the previously suggested DHB [22], [34], [35]. To the best of our knowledge, DD have not been previously analyzed by MALDI MS. NaDHB ionized easily TG giving [M+Na]^+^ molecular adducts, similarly like DHB, MBT and THAP, suggested by other authors [36]–[38]. The overview of performance of the matrices is given in Table S2.

For further data processing, the intensities of the MALDI MS peaks corresponding to molecular adduct were converted into relative percentages. As only lipids of the same lipid class were ionized during the MALDI MS analysis, the signal suppression by other components was considered negligible. The peak intensities were not corrected by any response factors.

### Data treatment

The chemical diversity and sex-specificity of the VC samples were evaluated using principal component analysis (PCA) and redundancy analysis (RDA) performed in the Canoco 4.5 package (Biometrics, Plant Research). The intensities of the MALDI-TOF MS responses for particular lipids within each lipid class were converted into relative percentages and the diversity of their quantitative patterns visualized using PCA. Subsequently, RDA analyses of standardized variables with sex as a categorical predictor and a Monte Carlo permutation test (unrestricted permutations, *n* = 999) were performed in order to test the significance of the differences between the relative patterns in the two sexes. Six selected TG and six selected WE with an important contribution to the differences between the two sexes were further fragmented and the relative intensities of their dominant fragments treated using the same approach. The relative proportions of 167 FAME obtained from the hydrolyzed VC lipids were arcsine transformed and subjected to PCA and RDA as described above. The differences corresponding to a p-value below 0.05 are reported as significant for the RDA and Monte Carlo permutation tests.

## Results and Discussion

### GC/MS of VC fatty acids

**Figure 1:**
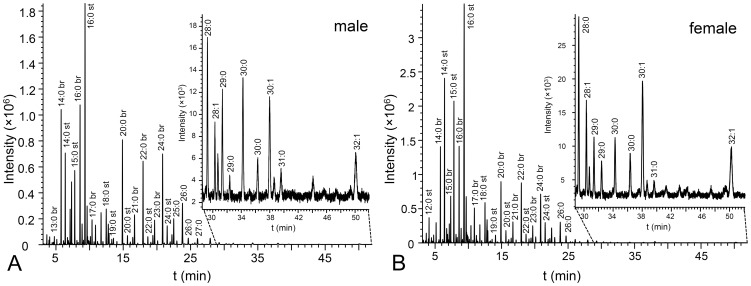
Chromatograms of the total lipid FAME. Characteristic reconstructed chromatogram (*m/z* 74) of FAME obtained by transesterification of vernix caseosa total lipid extract of a newborn boy (A) and girl (B).

**Figure 2:**
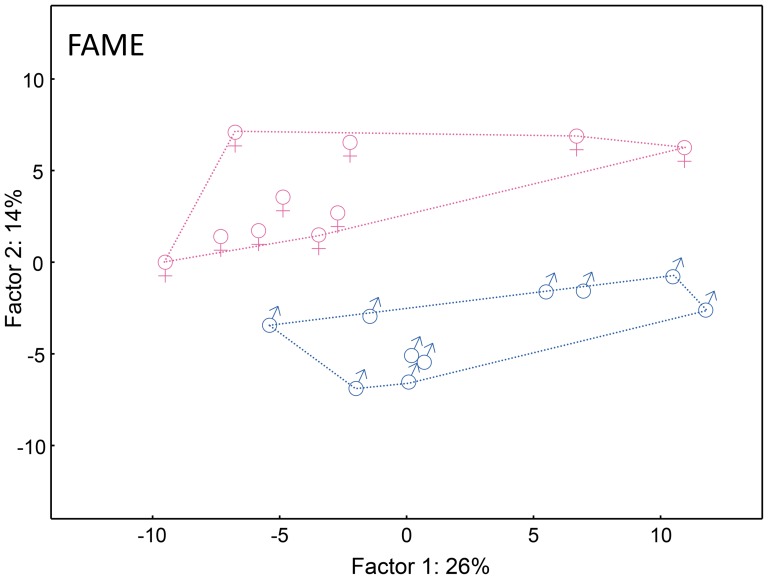
Quantitative pattern of vernix caseosa fatty acids in newborn boys (♂) and girls (♀). Graphic representation of the first two components of PCA calculated from the relative intensities of fatty acid methyl esters obtained from hydrolyzed vernix caseosa lipids.

**Figure 3:**
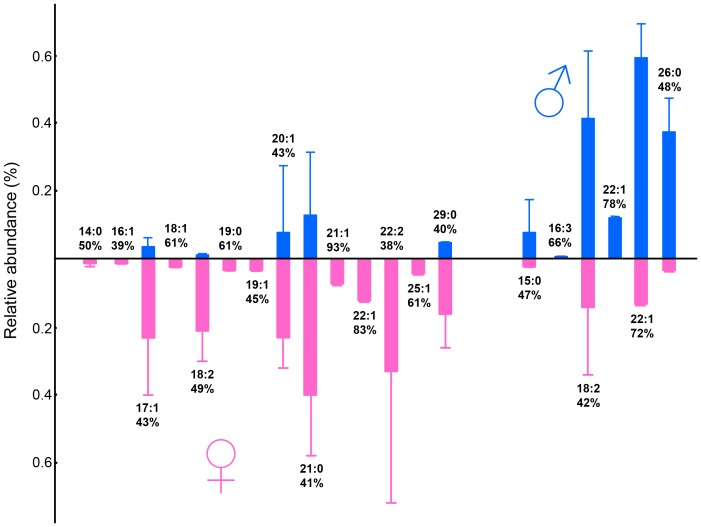
Comparison of relative abundances of fatty acid methyl esters from hydrolyzed vernix caseosa lipids obtained from newborn boys and girls. Relative abundances were calculated from peak areas in gas chromatograms (mean ± SD). Twenty compounds contributing the most to the sex-related differences are shown. The percentages below the peak assignments indicate the percent fit of individual variables with the model predictions of RDA with sex standing as categorical predictor.

Using a set of samples of 20 newborn subjects we investigated variability of VC lipids at the fatty acids level. We detected 167 distinct FAME species, mostly with saturated and branched chains, which is in agreement with recent report [39] showing 133 FAME in VC. FAME contained 11–31 carbons and exceptionally up to 4 double bonds (Table S3). Representative chromatograms are shown in Figure 1. When carefully inspecting chromatograms and peak lists, minor differences between boy and girl data were noticed. Visualization by means of PCA (Figure 2) using the first two principal components clearly showed that the samples were separated into two groups according to the sex of newborns. A redundancy analysis confirmed that the patterns of the relative abundances of FAME were significantly different between the male and female samples (F = 3.2; p = 0.002). The contributions of individual FAME to the observed overall differences are listed in the Table S3 as percent fits of each compound with the predicted RDA model with sex as categorical predictor. The sex specificity of the FAME composition consisted in both qualitative and quantitative differences in relative abundances. Among the FAME fitting the best the RDA model, monoenic or saturated species with typically more than 20 carbons occurred, but some middle-chain FAME with 14–19 carbons were also involved in sex differentiation (Figure 3 and Table S3). The most important species in this respect were FAME 21∶1 (peak No. 116) and FAME 22∶1 (peak No. 123) detected in non-negligible quantities only in the girl and boy samples, respectively. Nevertheless, the sex-related differences could not have been reduced to a list of only a few important species, the differences in quantitative patterns being complex. Encouraging results with hydrolyzed total lipid extracts showing differences between male and female subjects prompted us to study the chemical composition of intact lipids in boy and girl samples.

### MALDI MS of intact VC lipids

All lipid fractions obtained from VC samples provided rich MALDI spectra with series of peaks. The spectra of the SQ zone were an exception, as only a single signal of squalene was present. It is important to note that in general the peaks in the spectra could represent mixtures of lipid species having the same elemental composition. Like in all direct MS approaches (without chromatographic separation), the isomeric species cannot be distinguished by mass. Therefore, each peak was characterized by the total number of carbons and double bonds in the chains. An inspection of the mass spectra did not reveal any qualitative gender-related differences in the lipid composition of the studied fractions.

**Figure 4:**
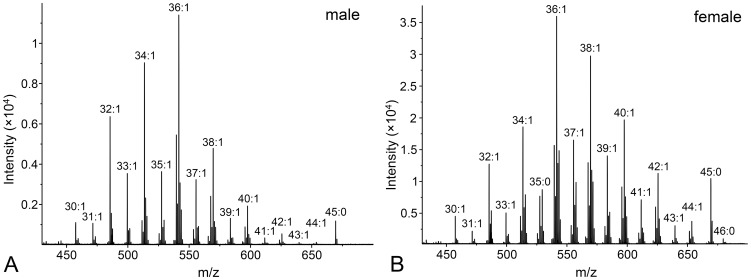
Mass spectra of the wax esters. Characteristic MALDI spectrum of the wax esters isolated from the vernix caseosa of a newborn boy (A) and girl (B). A LiDHB matrix was used and the signals correspond to molecular adducts with lithium ions [M+Li]^+^.

**Figure 5:**
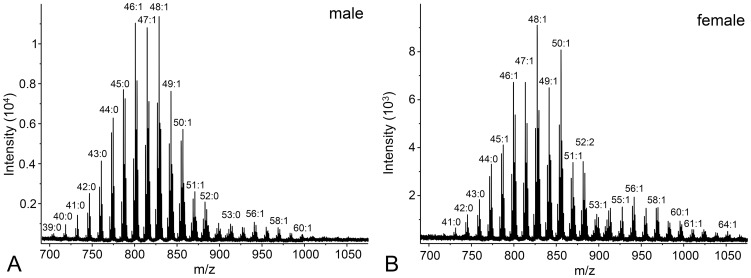
Mass spectra of the triacylglycerols. Characteristic MALDI spectrum of the triacylglycerols isolated from the vernix caseosa of a newborn boy (A) and girl (B). A NaDHB matrix was used and the signals correspond to molecular adducts with sodium ions [M+Na]^+^.

In the WE fraction (Figure 4 and Table S4), we observed wax esters with 26–46 carbons and up to three unsaturations; the most prominent peaks corresponded to molecules with one double bond in the chains. The CE fraction contained a series of cholesteryl esters with 14–32 carbons in the FA chain and between zero and two double bonds. In the DD fraction, we detected diol diesters with 46–64 carbon atoms, containing up to three unsaturations. TG with 39–69 carbons in the FA chains and with up to four double bonds were detected in the TG fraction (Figure 5 and Table S5). Dominant peaks represented molecules with either one or two unsaturations.

### Sex-related differences in intact lipids

Quantitative patterns of the relative intensities of particular compounds in each studied class (except for SQ) were compared using PCA and RDA with sex as a categorical predictor. The quantitative diversities within the DD and CE fractions were broadly overlapping in the two sexes and no significant gender-related differences could have been proved using RDA and Monte Carlo permutation tests.

**Figure 6:**
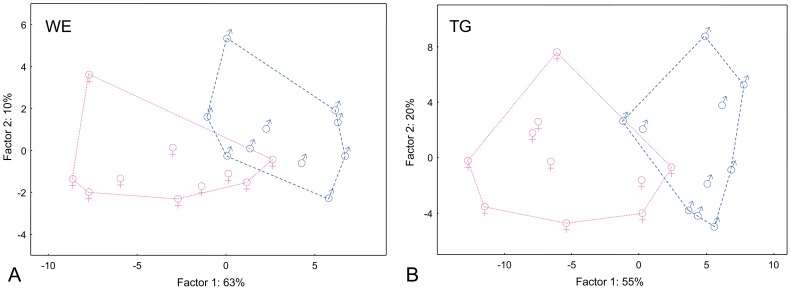
Quantitative pattern of vernix caseosa lipids in newborn boys and girls. Graphic representation of the first two components of PCA calculated from the relative intensities of the wax esters (A) and triacylglycerols (B) isolated from the vernix caseosa of newborn boys (♂) and girls (♀).

On the contrary, even a simple visual inspection of the mass spectra of WE and TG fractions made it possible to discriminate between the spectra of boys and girls (Figures 4 and 5). In both classes, the compounds with a higher carbon number seemed to be over-represented in female samples while shorter carbon chains were relatively more abundant in the spectra of males. When visualized by means of PCA, depicted in Figure 6 as the first two principal components of the WE and TG samples, the two sexes were separated into two slightly overlapping groups. A redundancy analysis confirmed that the pattern of the relative abundances of WE was significantly different between the male and female samples (F = 6.9; p = 0.008). The contributions of individual WE to the observed overall differences are listed in the Table S4 as percent fits of each compound with the predicted RDA model with sex as categorical predictor. The WE with higher chain lengths proved to be relatively over-represented in females, and vice versa, the short-chain WE were relatively more abundant in males. Similar conclusions were drawn for TG. The overall pattern of relative intensities differed significantly between males and females (F = 8.8; p = 0.002). Higher chain lengths were relatively more abundant in females while the relative proportions of TG were shifted towards shorter chain lengths in males, as shown in the Table S5.

### Fragmentation spectra of WE and TG

In light of these results, as several isomers can be found at the same *m/z* values, a question has arisen as to whether the observed differences in the WE and TG relative intensities reflect qualitative differences in the constituents of these WE and TG in boys and girls or rather quantitative differences in their production or selective sex-dependent incorporation of particular FA. To answer this question, we further fragmented twelve peaks from those most significantly contributing to the sex-specificity of TG and WE profiles and studied their identity and relative intensities of fragments in all samples using MALDI-TOF/TOF MS. Subsequently, the sex-specificity in the relative proportions of particular fragments in each fragmented compound was once again tested by means of RDA.

In the case of WE, the fragmentation spectra showed lithiated fatty acids originating from the acid parts of esters [26]. The spectra were qualitatively identical in all of the six peaks (WE 32:1, WE 34:1, WE 36:2, WE 40:1, WE 41:1, WE 42:1) and both sexes; the spectra were dominated by five signals representing over 95% of the total intensity, i.e. [FA 14:1+Li]^+^, [FA 15:0+Li]^+^, [FA 16:1+Li]^+^, [FA 17:1+Li]^+^ and [FA 18:1+Li]^+^. On the other hand, a RDA revealed significant gender-related differences in the relative intensities of these five fragments in all six fragmented peaks. Among the fatty acids contributing the most to the sex-related differences, the relative intensities of the fragments [FA 16:1+Li]^+^ and [FA 18:1+Li]^+^ were systematically over-represented in male and female subjects, respectively, with 37–75% fit with the predicted model for [FA 16:1+Li]^+^ and 36–74% fit for [FA 18:1+Li]^+^.

The fragmentation spectra of the six TG peaks (sodium adducts of TG 45:0, TG 45:1, TG 46:1, TG 52:1, TG 62:1, TG 64:1) showed signals consistent with neutral loss of fatty acids and fatty acid sodium salts. The fragments appeared in clusters differing from each other by the number of carbons. The most intense peak of each cluster corresponding to neutral loss of fatty acid sodium salt (Table 1) has been chosen for further study. There were no qualitative differences in the dominant fragments between the two sexes. However, like for WE, we detected significant differences between males and females in the relative proportions of the dominant fragments of the six fragmented TG.

**Table 1:** MALDI-TOF/TOF data for VC triacylglycerols.

Precursor [M+Na]^+^	Main fragments (*m/z*)	Neutral loss (RCOONa)
TG 45:1 (*m/z* 785.7)	481, 495, 509, 521, 535, 549, 563	FA 18:1, FA 17:1, FA 16:1, FA 15:0, FA 14:0, FA 13:0, FA 12:0
TG 45:0 (*m/z* 787.7)	481, 495, 509, 523, 537, 551, 565	FA 18:0, FA 17:0, FA 16:0, FA 15:0, FA 14:0, FA 13:0, FA 12:0
TG 46:1 (*m/z* 799.7)	495, 509, 523, 535, 549, 563, 577	FA 18:1, FA 17:1, FA 16:1, FA 15:0, FA 14:0, FA 13:0, FA 12:0
TG 52:1 (*m/z* 883.8)	495, 509, 523, 535, 549, 563, 577	FA 24:1, FA 23:1, FA 22:1, FA 21:0, FA 20:0, FA 19:0, FA 18:0
TG 62:1 (*m/z* 1023.9)	523, 549, 577, 605, 717, 745, 773	FA 32:1, FA 30:0, FA 28:0, FA 26:0, FA 18:0, FA 16:0, FA 14:0
TG 64:1 (*m/z* 1051.8)	523, 551, 577, 605, 745, 773, 801	FA 34:1, FA 32:1, FA 30:0, FA 28:0, FA 18:0, FA 16:0, FA 14:0

### Sex-specificity of VC lipid composition

Our results strongly support the hypothesis that the composition of VC lipids is gender-related. We showed statistically significant differences between male and female samples both at the level of fatty acids in the total lipid extracts and at the level of intact lipids in two lipid classes. At the current stage of our knowledge, we can only hypothesize the biological aspects underlying these differences. First, the differences in VC chemistry may result from differential temporal dynamics in the skin development in boys and girls controlled by steroid hormones; previous studies in rats have documented that the formation of the cutaneous barrier is accelerated by estrogen and delayed by testosterone [40]. VC of human male fetuses was previously shown to contain more sebum than that of female fetuses, which has a higher proportion of epidermal lipids [15]. We found the differences in WE and TG, i.e., lipid classes that are of sebaceous origin [1]. Therefore, the observed sex-related differences are likely associated with the activities of sebaceous glands in the skin of the fetus. Interestingly, when we analyzed VC obtained from a girl prematurely born in the 35^th^ week, the lipid profiles greatly differed from those of full-term girls and were rather similar to that of full-term boys. This accidental observation further supports the hypothesis of differential dynamics in VC production between the two sexes. Alternatively, permanent and fixed differences in the chemistry of the storage pool of FA, shifted towards longer carbon chains in some lipid classes in females, can account for the observed sex specificity of VC lipids.

The quest for an unambiguous verification of these hypotheses prompts further studies aiming at dynamics in VC production and composition involving newborn males and females of varied gestational age. Because of extreme complexity of VC lipids, lipidomics approaches based on cutting edge analytical chemistry are desirable.

## Conclusions

In the present study, we show that the quantitative pattern of lipids contained in the vernix caseosa of full-term newborns is sex-specific, namely because of the higher proportions of wax esters and triacylglycerols with longer hydrocarbon chains in newborn girls. These results pave the way to further investigations of the vernix caseosa, aiming at both structural and dynamic patterns of the lipid constituents and biological determinants underlying these patterns.

## Supporting Information

Figure S1 Image of semipreparative thin layer silica gel plate with separated zones of vernix caseosa lipids.
(PDF)

Table S1 List of subjects, their basic biological characteristics and sampled body parts.
(PDF)

Table S2 Suitability of the MALDI matrices for neutral lipids of vernix caseosa.
(PDF)

Table S3 Relative peak areas of fatty acid methyl esters.
(PDF)

Table S4 Relative intensities of wax esters in vernix caseosa of newborn boys and girls.
(PDF)

Table S5 Relative intensities of triacylglycerols in vernix caseosa of newborn boys and girls calculated from MALDI spectra (mean ± SD).
(PDF)
