# Correction: Newborn Boys and Girls Differ in the Lipid Composition of Vernix Caseosa

**DOI:** 10.1371/journal.pone.0107847

**Published:** 2014-09-08

**Authors:** The PLOS ONE Staff

There are errors in the Funding Statement. The correct funding is as follows: “Funding for this project was provided by Czech Science Foundation (Project No. 206/12/0750), Academy of Sciences of the Czech Republic (Project RVO 61388963) and Charles University in Prague (Project SVV 260084). The funders had no role in study design, data collection and analysis, decision to publish, or preparation of the manuscript.”
