# Supplementary material for: Newborn Boys and Girls Differ in the Lipid Composition of Vernix Caseosa
Source: PLoS One. 2014 Jun 9;9(6):e99173. doi: 10.1371/journal.pone.0099173 (PMC4049714; doi:10.1371/journal.pone.0099173)
Supplement: Figure S1 — Image of semipreparative thin layer silica gel plate with separated zones of vernix caseosa lipids. (PDF) [file pone.0099173.s001.pdf]

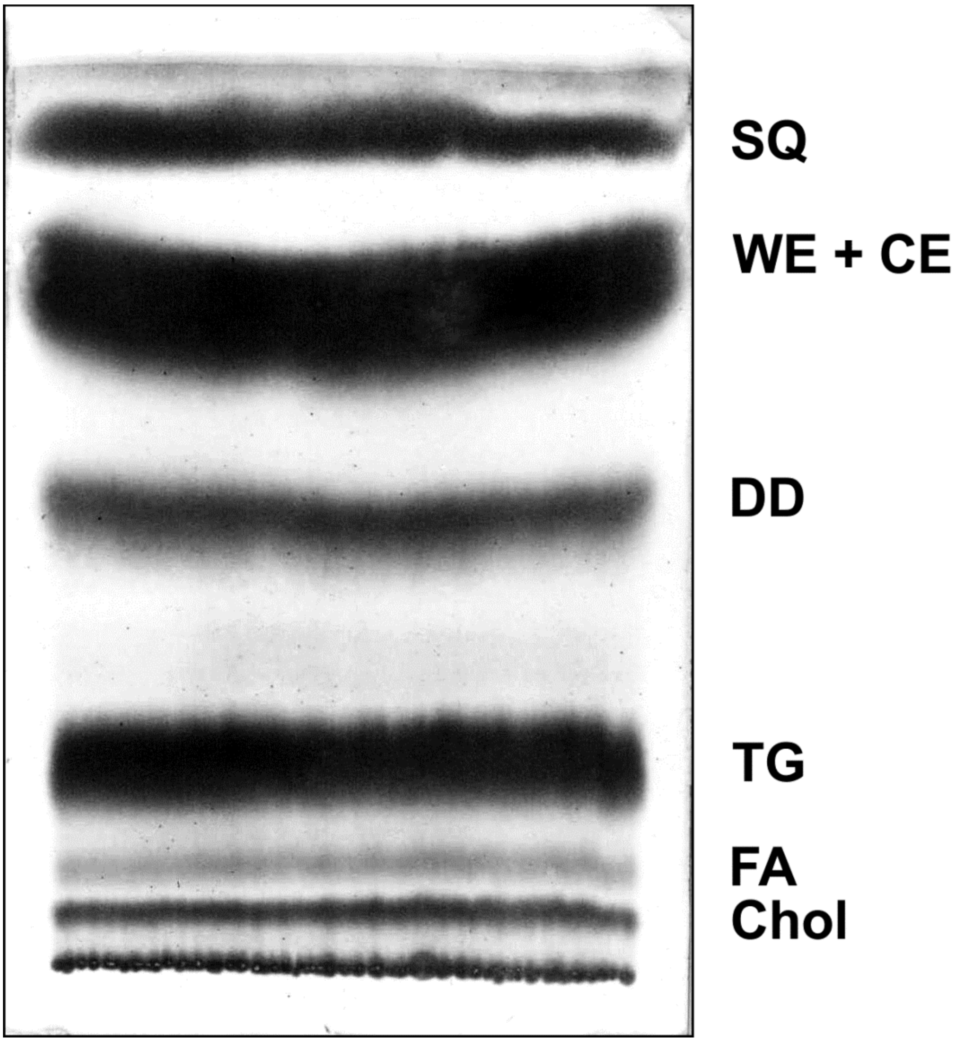

Figure S1. Image of semipreparative 9 x 12 cm thin layer silica gel plate with separated zones of VC lipids (SQ: squalene; WE: wax esters; CE: cholesteryl esters; DD: diol diesters; TG: triacylglycerols; FA: free fatty acids; Chol: sterols).
