# Supplementary material for: Newborn Boys and Girls Differ in the Lipid Composition of Vernix Caseosa
Source: PLoS One. 2014 Jun 9;9(6):e99173. doi: 10.1371/journal.pone.0099173 (PMC4049714; doi:10.1371/journal.pone.0099173)
Supplement: Table S1 — List of subjects, their basic biological characteristics and sampled body parts. (PDF) [file pone.0099173.s002.pdf]

Table S1. List of subjects, their basic biological characteristics and sampled body parts.

No.	Sex	Delivery	Sampling part	Length (cm)	Weight (g)
1	♀	Caesarean section	back	50	3520
2	♂	vaginal	back	50	3115
3	♀	vaginal	back, buttocks	48	3000
4	♂	vaginal	groins	50	3320
5	♂	vaginal	back	45	2510
6	♀	Caesarean section	back	49	3640
7	♂	vaginal	leg	49	3190
8	♂	Caesarean section	arm	50	2770
9	♀	vaginal	back	53	3850
10	♀	vaginal	back	51	3410
11	♂	Caesarean section	leg	50	3450
12	♂	Caesarean section	back	53	4110
13	♀	vaginal	buttocks	52	3690
14	♂	Caesarean section	back	53	3090
15	♀	Caesarean section	back, leg	52	3800
16	♂	Caesarean section	leg	48	3670
17	♂	vaginal	back	52	3220
18	♀	vaginal	back	49	3120
19	♀	vaginal	back	49	3120
20	♀	vaginal	buttocks	49	3160
