# Supplementary material for: Newborn Boys and Girls Differ in the Lipid Composition of Vernix Caseosa
Source: PLoS One. 2014 Jun 9;9(6):e99173. doi: 10.1371/journal.pone.0099173 (PMC4049714; doi:10.1371/journal.pone.0099173)
Supplement: Table S2 — Suitability of the MALDI matrices for neutral lipids of vernix caseosa. (PDF) [file pone.0099173.s003.pdf]

Table S2. Suitability of the MALDI matrices for neutral lipids of vernix caseosa.

Lipids	Quality of the signal [a],[b]							
	DHB	NaDHB	LiDHB	MBT	TCNQ	4NA	PA	THAP
SQ	++	++	+++		++		++	
WE		++	+++			+		
CE	+	++	+++			+		
DD		+++	+++					
TG	+++	+++		+++	++	+	++	++

[a]) +++ excellent signal quality, no interference with matrix ions; ++ good signal quality, interferences with matrix ions; + poor signal quality

[b]) solvents used: methanol (DHB, MBT, 4NA); acetone (NaDHB); acetone/chloroform 2:1 (LiDHB); toluene (TCNQ); water/acetonitrile 2:1 + 0.1%TFA (PA); water/acetonitrile 1:1 + 0.1%TFA (THAP)
