# Supplementary material for: Newborn Boys and Girls Differ in the Lipid Composition of Vernix Caseosa
Source: PLoS One. 2014 Jun 9;9(6):e99173. doi: 10.1371/journal.pone.0099173 (PMC4049714; doi:10.1371/journal.pone.0099173)
Supplement: Table S3 — Relative peak areas of fatty acid methyl esters. (PDF) [file pone.0099173.s004.pdf]

Table S3. Relative peak areas of fatty acid methyl esters (FAME) obtained by hydrolysis of vernix caseosa lipids and calculated from GC/EI-MS data (mean± SD).

% fit states the correspondence of individual variables with the model prediction on sex specificity of relative intensities extracted from RDA analysis.

FAME annotations: br – branched; st – straight chain.

Peak No.	1	2	3	4	5	6	7	8	9	10	11	12	13	14	15	16	17	18	19	20
FAME	11:0 br	11:0 st	12:0 br	12:0 br	12:0 br	12:0 br	13:0 br	12:0 st	13:0 br	13:0 br	13:0 br	13:0 br	13:0 br	13:0 st	13:1	14:0 br	14:0 br	14:0 br	14:0 br	14:0 st
♂	0.01 ± 0.01	0.04 ± 0.02	0.01 ± 0.00	0.04 ± 0.03	0.21 ± 0.09	0.05 ± 0.04	0.20 ± 0.13	0.25 ± 0.11	0.04 ± 0.03	0.14 ± 0.09	0.06 ± 0.03	0.05 ± 0.02	0.29 ± 0.05	1.00 ± 0.78	0.06 ± 0.06	0.11 ± 0.08	0.01 ± 0.00	2.98 ± 0.77	0.01 ± 0.01	0.00 ± 0.00
% fit	<30	<30	<30	<30	<30	<30	<30	<30	<30	<30	<30	<30	<30	<30	<30	<30	<30	<30	<30	50
♀	0.00 ± 0	0.03 ± 0	0.01 ± 0	0.02 ± 0	0.15 ± 0.1	0.03 ± 0	0.10 ± 0.1	0.22 ± 0.2	0.03 ± 0	0.12 ± 0.1	0.05 ± 0	0.06 ± 0	0.24 ± 0.1	1.57 ± 1.2	0.04 ± 0	0.07 ± 0	0.01 ± 0	2.75 ± 0.6	0.01 ± 0	0.01 ± 0.01

Peak No.	21	22	23	24	25	26	27	28	29	30	31	32	33	34	35	36	37	38	39	40
FAME	14:1	15:0 br	15:0 br	15:0 br	14:0 st	15:0 br	14:1	14:1	14:1	15:0 br	15:0 br	15:0 br	14:1	15:0 br	15:0 br	15:0 br	15:1	16:0 br	16:0 br	16:0 br
♂	0.02 ± 0.01	0.14 ± 0.1	0.08 ± 0.1	0.00 ± 0	3.2 ± 1.1	0.01 ± 0	0.01 ± 0	0.96 ± 0.5	0.13 ± 0.1	0.24 ± 0.2	0.21 ± 0.2	0.07 ± 0.1	0.79 ± 2.5	0.73 ± 0.2	0.05 ± 0	2.38 ± 0.5	0.15 ± 0.1	0.01 ± 0	0.01 ± 0	0.02 ± 0.01
% fit	30	31	47	30	<30	<30	<30	<30	<30	<30	<30	31	<30	<30	<30	<30	<30	<30	<30	<30
♀	0.05 ± 0	0.06 ± 0	0.02 ± 0	0.01 ± 0	2.80 ± 1.5	0.00 ± 0	0.00 ± 0	0.79 ± 0.5	0.06 ± 0	0.30 ± 0.2	0.22 ± 0.3	0.30 ± 0.2	0.13 ± 0.2	1.7 ± 0.7	0.05 ± 0	2.49 ± 0.7	0.14 ± 0.1	0.00 ± 0	0.01 ± 0	0.01 ± 0.01

Peak No.	41	42	43	44	45	46	47	48	49	50	51	52	53	54	55	56	57	58	59	60
FAME	15:0 st	15:1	15:1	16:0 br	16:0 br	16:0 br	16:0 br	16:1	17:0 br	17:0 br	16:0 br	17:0 br	16:0 st	16:1	16:1	16:1 st	17:0 br	17:0 br	17:0 br	17:0 br
♂	3.33 ± 1.50	0.13 ± 0.24	0.78 ± 0.46	0.11 ± 0.07	0.29 ± 0.21	4.26 ± 1.4	0.00 ± 0.00	2.15 ± 1.3	0.12 ± 0.07	0.29 ± 0.16	0.00 ± 0.00	0.09 ± 0.08	10.7 ± 2.8	0.00 ± 0.00	0.01 ± 0.01	6.22 ± 2.89	1.39 ± 0.41	0.55 ± 0.30	0.33 ± 0.22	0.26 ± 0.16
% fit	<30	<30	<30	<30	<30	<30	<30	<30	<30	<30	<30	<30	<30	39	<30	31	<30	<30	<30	<30
♀	2.83 ± 1.2	0.03 ± 0	0.58 ± 0.3	0.07 ± 0	0.17 ± 0.1	4.46 ± 1	0.01 ± 0	1.48 ± 0.9	0.14 ± 0.2	0.21 ± 0.2	0.07 ± 0.1	1.31 ± 2.6	9.18 ± 4.1	0.01 ± 0	2.16 ± 4.1	3.16 ± 2.2	1.4 ± 0.9	0.40 ± 0.2	0.44 ± 0.2	0.15 ± 0.12

Peak No.	61	62	63	64	65	66	67	68	69	70	71	72	73	74	75	76	77	78	79	80
FAME	17:0 br	16:1	17:0 br	17:1	17:0 br	16:2	17:1	17:0 st	17:1	17:1 st	17:1	17:1	18:0 br	18:1	18:1	18:0 st	16:3	18:1	18:1 st	18:1 st
♂	0.05 ± 0.1	0.13 ± 0.1	0.11 ± 0.1	0.53 ± 0.3	0.87 ± 0.4	0.05 ± 0.1	0.41 ± 0.4	1.00 ± 0.4	0.90 ± 0.5	0.32 ± 0.1	0.12 ± 0.2	0.07 ± 0	0.92 ± 0.3	0.62 ± 0.2	0.02 ± 0	1.48 ± 0.2	0.01 ± 0	0.00 ± 0	6.21 ± 1.1	2.27 ± 0.69
% fit	<30	<30	<30	31	<30	<30	<30	<30	<30	<30	<30	43	<30	<30	<30	<30	66	61	<30	<30
♀	0.01 ± 0	0.12 ± 0.1	0.21 ± 0.2	0.06 ± 0	0.98 ± 0.5	0.07 ± 0.1	0.56 ± 0.2	0.93 ± 0.3	0.40 ± 0.4	0.56 ± 0.2	0.20 ± 0.2	0.20 ± 0	1.00 ± 0.3	0.52 ± 0.3	0.17 ± 0.1	1.60 ± 0.3	0.00 ± 0	0.02 ± 0	6.13 ± 0.4	2.88 ± 0.85

Peak No.	81	82	83	84	85	86	87	88	89	90	91	92	93	94	95	96	97	98	99	100
FAME	18:2	18:2	18:2	19:0 br	18:2	18:2 st	18:2 st	18:2	19:1	19:0 br	19:0 st	19:1	19:1	19:1	19:1	19:1	20:0 br	20:1	0,875	21:0 br
♂	0.42 ± 0.2	0.01 ± 0	0.00 ± 0	0.20 ± 0.1	0.39 ± 0.1	1.22 ± 0.5	0.24 ± 0.1	0.05 ± 0.1	0.02 ± 0	0.00 ± 0	0.24 ± 0.1	0.17 ± 0.1	0.09 ± 0.1	0.03 ± 0	0.03 ± 0	0.00 ± 0	3.78 ± 1.1	0.33 ± 0.1	0.11 ± 0.1	0.01 ± 0.02
% fit	42	49	<30	<30	<30	<30	<30	<30	<30	61	<30	<30	<30	<30	<30	45	<30	<30	<30	<30
♀	0.14 ± 0.2	0.21 ± 0.1	0.04 ± 0.1	0.20 ± 0.1	0.47 ± 0.2	1.2 ± 0.4	0.26 ± 0.1	0.04 ± 0.1	0.03 ± 0.1	0.03 ± 0	0.19 ± 0.1	0.17 ± 0.1	0.14 ± 0.1	0.08 ± 0.1	0.03 ± 0	0.03 ± 0	4.10 ± 1.3	0.39 ± 0.1	0.04 ± 0	0.05 ± 0.06

Peak No.	101	102	103	104	105	106	107	108	109	110	111	112	113	114	115	116	117	118	119	120
FAME	21:0 br	20:0 st	20:1 st	20:1	20:1	20:1	20:1	21:0 br	20:2	21:0 br	20:3 st	21:0 st	21:1	20:4 st	21:1	21:1	22:0 br	22:1	22:0 br	22:0 br
♂	0.00 ± 0	0.61 ± 0.1	0.19 ± 0.1	0.08 ± 0.2	0.71 ± 0.2	0.13 ± 0.1	0.20 ± 0.2	0.13 ± 0.2	0.05 ± 0.1	0.74 ± 0.5	0.98 ± 0.4	0.11 ± 0.1	0.13 ± 0.4	0.07 ± 0.1	0.03 ± 0.1	0.00 ± 0	4.02 ± 0.9	0.00 ± 0	0.17 ± 0.2	0.07 ± 0.03
% fit	<30	<30	<30	43	<30	33	<30	41	30	35	31	<30	<30	<30	<30	93	<30	83	<30	<30
♀	0.01 ± 0	0.73 ± 0.1	0.18 ± 0.2	0.23 ± 0.1	0.28 ± 0.2	0.78 ± 0.6	0.16 ± 0.1	0.40 ± 0.2	0.12 ± 0.1	1.79 ± 0.5	0.07 ± 0.1	0.20 ± 0.2	0.13 ± 0.3	0.09 ± 0	0.06 ± 0	0.07 ± 0	4.38 ± 1.2	0.12 ± 0	0.21 ± 0.2	0.07 ± 0.05

Peak No.	121	122	123	124	125	126	127	128	129	130	131	132	133	134	135	136	137	138	139	140
FAME	22:0 st	22:1	22:1 st	22:1	22:1	23:0 br	22:2	22:2	23:0 st	23:0 st	24:0 br	24:0 br	23:1	24:0 br	24:0 st	24:1 st	24:1 st	25:0	25:0	25:0
♂	**0.45 ± 0.1**	**0.12 ± 0**	**0.60 ± 0.1**	**0.79 ± 0.4**	**0.21 ± 0.1**	**0.40 ± 0.1**	**0.02 ± 0**	**0.00 ± 0**	**1.32 ± 0.4**	**0.26 ± 0.1**	**3.61 ± 1.6**	**0.07 ± 0.1**	**0.17 ± 0.1**	**0.16 ± 0.1**	**1.23 ± 0.1**	**0.39 ± 0.1**	**0.73 ± 0.4**	**0.28 ± 0.1**	**0.22 ± 0.2**	**0.94 ± 0.84**
% fit	<30	**78**	**72**	<30	<30	<30	<30	**38**	<30	<30	<30	<30	<30	<30	<30	<30	<30	<30	**34**	<30
♀	**0.47 ± 0.2**	**0.00 ± 0**	**0.13 ± 0**	**1.15 ± 0.4**	**0.20 ± 0.1**	**0.35 ± 0.2**	**0.08 ± 0.1**	**0.33 ± 0.4**	**1.07 ± 0.8**	**0.26 ± 0.1**	**4.01 ± 1.1**	**0.08 ± 0.1**	**0.25 ± 0.2**	**0.10 ± 0.1**	**1.29 ± 0.3**	**0.41 ± 0.2**	**0.93 ± 0.5**	**0.13 ± 0**	**0.48 ± 0.2**	**0.96 ± 0.92**

Peak No.	141	142	143	144	145	146	147	148	149	150	151	152	153	154	155	156	157	158	159	160
FAME	25:0	25:0	26:0	25:1	26:0	26:0	26:0	26:0	26:0	26:0	26:1	27:0	27:0	27:0	28:0	28:0	28:1	29:0	29:0	29:0
♂	**0.69 ± 1**	**0.32 ± 0.1**	**0.26 ± 0.5**	**0.00 ± 0**	**2.64 ± 0.6**	**0.28 ± 0.2**	**0.20 ± 0.2**	**0.38 ± 0.1**	**0.79 ± 0.2**	**0.38 ± 0.1**	**0.33 ± 0.1**	**0.29 ± 0.1**	**0.66 ± 0.3**	**0.11 ± 0**	**0.59 ± 0.2**	**0.27 ± 0.1**	**0.45 ± 0.2**	**0.05 ± 0**	**0.17 ± 0.1**	**0.05 ± 0.02**
% fit	<30	<30	<30	**61**	<30	**31**	<30	**48**	<30	**32**	<30	<30	<30	<30	<30	<30	<30	**40**	<30	<30
♀	**0.89 ± 0.8**	**0.39 ± 0.3**	**0.03 ± 0.1**	**0.04 ± 0**	**2.56 ± 0.8**	**0.00 ± 0**	**0.11 ± 0.3**	**0.03 ± 0**	**0.87 ± 0.2**	**0.07 ± 0.2**	**0.45 ± 0.2**	**0.25 ± 0.2**	**0.57 ± 0.3**	**0.12 ± 0**	**0.57 ± 0.2**	**0.38 ± 0.2**	**0.65 ± 0.2**	**0.16 ± 0.1**	**0.21 ± 0.1**	**0.09 ± 0.09**

Peak No.	161	162	163	164	165	166	167
FAME	30:0	30:0	30:1	31:0	31:0	31:0	32:1
♂	**0.25 ± 0.3**	**0.24 ± 0.4**	**0.96 ± 0.6**	**0.05 ± 0.1**	**0.22 ± 0.4**	**0.00 ± 0**	**0.58 ± 0.3**
% fit	<30	<30	<30	<30	<30	**37**	<30
♀	**0.16 ± 0.1**	**0.27 ± 0.5**	**1.12 ± 0.5**	**0.07 ± 0.1**	**0.18 ± 0.4**	**0.01 ± 0**	**0.70 ± 0.3**
