# Supplementary material for: Newborn Boys and Girls Differ in the Lipid Composition of Vernix Caseosa
Source: PLoS One. 2014 Jun 9;9(6):e99173. doi: 10.1371/journal.pone.0099173 (PMC4049714; doi:10.1371/journal.pone.0099173)
Supplement: Table S4 — Relative intensities of wax esters in vernix caseosa of newborn boys and girls. (PDF) [file pone.0099173.s005.pdf]

Table S4. Relative intensities of wax esters in vernix caseosa of newborn boys and girls calculated from MALDI spectra (mean ± SD).

% fit states the correspondence of individual variables with the model prediction on sex specificity of relative intensities extracted from RDA analysis.

WE:	30:1	30:0	31:1	31:0	32:1	32:0	33:1	33:0	34:2	34:1	34:0	35:2	35:1	35:0	36:2	36:1	36:0	37:2	37:1	37:0	38:2
♂	**1.79 ± 0.50**	**0.32 ± 0.14**	**1.30 ± 0.52**	**0.48 ± 0.19**	**7.20 ± 1.95**	**0.91 ± 0.27**	**4.04 ± 1.18**	**0.81 ± 0.25**	**1.68 ± 0.37**	**10.34 ± 2.97**	**1.84 ± 0.43**	**0.72 ± 0.31**	**4.53 ± 1.29**	**1.95 ± 0.61**	**6.84 ± 1.32**	**13.32 ± 2.99**	**2.77 ± 0.90**	**1.04 ± 0.37**	**5.30 ± 0.89**	**1.92 ± 0.84**	**3.66 ± 0.59**
% fit	<30	<30	<30	<30	**47**	<30	**48**	<30	<30	**46**	<30	<30	**31**	<30	**42**	**36**	<30	<30	<30	<30	<30
♀	**1.44 ± 0.96**	**0.24 ± 0.13**	**1.03 ± 0.7**	**0.31 ± 0.17**	**3.98 ± 2.54**	**0.59 ± 0.33**	**2.39 ± 1.6**	**0.55 ± 0.21**	**1.30 ± 0.78**	**6.27 ± 3.26**	**1.78 ± 0.53**	**0.66 ± 0.37**	**3.43 ± 1.43**	**2.26 ± 0.57**	**4.66 ± 2.1**	**10.09 ± 2.44**	**3.42 ± 0.98**	**0.98 ± 0.36**	**5.57 ± 0.94**	**2.58 ± 0.87**	**3.66 ± 0.45**

WE:	38:1	38:0	39:2	39:1	39:0	40:2	40:1	40:0	41:2	41:1	41:0	42:2	42:1	42:0	43:2	43:1	43:0	44:2	44:1	44:0
♂	**7.47 ± 1.24**	**1.78 ± 0.85**	**0.55 ± 0.19**	**2.95 ± 0.81**	**1.03 ± 0.63**	**1.80 ± 0.54**	**3.86 ± 1.36**	**0.89 ± 0.65**	**0.21 ± 0.08**	**1.27 ± 0.76**	**0.47 ± 0.49**	**0.76 ± 0.57**	**2.00 ± 1.42**	**0.36 ± 0.46**	**0.06 ± 0.04**	**0.58 ± 0.53**	**0.15 ± 0.26**	**0.25 ± 0.24**	**0.71 ± 0.72**	**0.08 ± 0.13**
% fit	<30	**32**	**49**	**43**	<30	**53**	**41**	<30	**60**	**44**	<30	**52**	**34**	<30	**44**	**30**	<30	**47**	<30	<30
♀	**8.97 ± 2.06**	**2.67 ± 1.05**	**0.90 ± 0.26**	**4.49 ± 1.39**	**1.73 ± 0.92**	**2.83 ± 0.81**	**6.01 ± 1.69**	**1.56 ± 1.03**	**0.54 ± 0.27**	**2.45 ± 1**	**0.78 ± 0.57**	**1.82 ± 0.87**	**3.72 ± 1.53**	**0.57 ± 0.47**	**0.20 ± 0.15**	**1.15 ± 0.66**	**0.21 ± 0.17**	**0.69 ± 0.42**	**1.37 ± 0.69**	**0.12 ± 0.11**
