# Supplementary material for: Newborn Boys and Girls Differ in the Lipid Composition of Vernix Caseosa
Source: PLoS One. 2014 Jun 9;9(6):e99173. doi: 10.1371/journal.pone.0099173 (PMC4049714; doi:10.1371/journal.pone.0099173)
Supplement: Table S5 — Relative intensities of triacylglycerols in vernix caseosa of newborn boys and girls calculated from MALDI spectra (mean ± SD). (PDF) [file pone.0099173.s006.pdf]

Table S5. Relative intensities of triacylglycerols in vernix caseosa of newborn boys and girls calculated from MALDI spectra (mean ± SD).

% fit states the correspondence of individual variables with the model prediction on sex specificity of relative intensities extracted from RDA analysis.

TG:	39:0	40:0	41:1	41:0	42:2	42:1	42:0	43:2	43:1	43:0	44:2	44:1	44:0	45:2	45:1	45:0	46:3	46:2	46:1	46:0	47:3
♂	0.23 ± 0.06	0.32 ± 0.10	0.29 ± 0.08	0.54 ± 0.14	0.16 ± 0.05	0.59 ± 0.16	1.07 ± 0.23	0.22 ± 0.06	1.10 ± 0.21	1.79 ± 0.28	0.50 ± 0.15	2.56 ± 0.23	3.06 ± 0.34	0.80 ± 0.19	3.67 ± 0.31	3.79 ± 0.64	0.24 ± 0.07	2.05 ± 0.30	6.29 ± 0.77	4.75 ± 0.77	0.28 ± 0.09
% fit	<30	<30	<30	<30	<30	<30	<30	<30	<30	<30	<30	<30	**43**	<30	**36**	**40**	<30	<30	**50**	**48**	<30
♀	0.21 ± 0.05	0.26 ± 0.09	0.27 ± 0.06	0.42 ± 0.19	0.18 ± 0.05	0.48 ± 0.21	0.74 ± 0.41	0.22 ± 0.06	0.84 ± 0.39	1.24 ± 0.59	0.43 ± 0.18	1.76 ± 0.83	2.10 ± 0.7	0.64 ± 0.29	2.61 ± 0.96	2.76 ± 0.61	0.21 ± 0.07	1.47 ± 0.62	4.34 ± 1.13	3.50 ± 0.47	0.24 ± 0.08

TG:	47:2	47:1	47:0	48:3	48:2	48:1	48:0	49:3	49:2	49:1	49:0	50:4	50:3	50:2	50:1	50:0	51:3	51:2	51:1	51:0	52:4
♂	2.23 ± 0.3	6.07 ± 1.04	4.16 ± 0.87	0.76 ± 0.2	3.91 ± 0.4	7.33 ± 1.1	3.69 ± 0.56	0.51 ± 0.14	2.39 ± 0.29	4.36 ± 0.55	1.98 ± 0.33	0.19 ± 0.05	1.00 ± 0.24	3.18 ± 0.56	4.24 ± 0.91	1.40 ± 0.26	0.45 ± 0.11	1.17 ± 0.16	1.40 ± 0.15	0.61 ± 0.12	0.24 ± 0.12
% fit	<30	**33**	<30	<30	**42**	<30	<30	<30	<30	<30	<30	<30	<30	<30	**36**	**32**	<30	**67**	**60**	**44**	<30
♀	1.67 ± 0.62	4.73 ± 0.79	3.41 ± 0.37	0.57 ± 0.24	2.90 ± 0.78	6.57 ± 0.89	3.52 ± 0.51	0.46 ± 0.17	2.28 ± 0.46	5.11 ± 0.74	2.21 ± 0.36	0.18 ± 0.05	0.88 ± 0.22	3.72 ± 0.62	6.41 ± 2.38	1.93 ± 0.48	0.53 ± 0.1	1.92 ± 0.34	2.45 ± 0.63	0.90 ± 0.21	0.23 ± 0.04

TG:	52:3	52:2	52:1	52:0	53:3	53:2	53:1	53:0	54:4	54:3	54:2	54:1	54:0	55:2	55:1	55:0	56:2	56:1	56:0	57:2	57:1
♂	0.82 ± 0.64	1.67 ± 1.17	1.18 ± 0.43	0.53 ± 0.16	0.21 ± 0.05	0.35 ± 0.06	0.45 ± 0.06	0.39 ± 0.09	0.21 ± 0.18	0.37 ± 0.37	0.42 ± 0.21	0.53 ± 0.12	0.51 ± 0.16	0.18 ± 0.04	0.39 ± 0.06	0.48 ± 0.13	0.23 ± 0.05	0.56 ± 0.14	0.59 ± 0.24	0.17 ± 0.04	0.40 ± 0.08
% fit	<30	<30	**44**	**34**	**51**	**59**	**62**	**40**	<30	<30	**32**	**51**	<30	**39**	**55**	**33**	**39**	**43**	<30	<30	**53**
♀	0.88 ± 0.15	2.74 ± 0.94	2.07 ± 0.56	0.79 ± 0.19	0.32 ± 0.08	0.65 ± 0.2	0.75 ± 0.17	0.58 ± 0.14	0.20 ± 0.04	0.45 ± 0.13	0.68 ± 0.16	0.80 ± 0.17	0.74 ± 0.2	0.26 ± 0.07	0.59 ± 0.15	0.75 ± 0.22	0.30 ± 0.09	0.83 ± 0.19	0.85 ± 0.31	0.23 ± 0.05	0.66 ± 0.18

TG:	57:0	58:2	58:1	58:0	59:2	59:1	59:0	60:2	60:1	60:0	61:2	61:1	61:0	62:2	62:1	62:0	63:1	64:2	64:1
♂	0.43 ± 0.12	0.20 ± 0.05	0.45 ± 0.17	0.39 ± 0.16	0.13 ± 0.03	0.27 ± 0.07	0.22 ± 0.07	0.15 ± 0.04	0.26 ± 0.12	0.21 ± 0.09	0.10 ± 0.03	0.15 ± 0.04	0.13 ± 0.03	0.10 ± 0.03	0.15 ± 0.06	0.13 ± 0.05	0.10 ± 0.03	0.08 ± 0.03	0.11 ± 0.04
% fit	**32**	**34**	**44**	<30	**45**	**53**	**38**	**39**	**43**	<30	**38**	**44**	<30	**44**	**40**	<30	**43**	**45**	**34**
♀	0.66 ± 0.22	0.28 ± 0.08	0.79 ± 0.23	0.60 ± 0.24	0.21 ± 0.06	0.52 ± 0.17	0.39 ± 0.13	0.24 ± 0.08	0.48 ± 0.19	0.33 ± 0.12	0.15 ± 0.05	0.29 ± 0.11	0.21 ± 0.08	0.19 ± 0.08	0.31 ± 0.12	0.19 ± 0.08	0.20 ± 0.08	0.17 ± 0.07	0.20 ± 0.09
